# Daily HIV pre-exposure prophylaxis enhances monocyte activation and reprogrammes immune cell metabolism

**DOI:** 10.3389/fimmu.2026.1910812

**Published:** 2026-08-14

**Authors:** Grainne Jameson, Dearbhla M. Murphy, Isabella Batten, Sarah A. Connolly, Adam H. Dyer, Annmarie White, Sarah Cochrane, Dearbhla Murphy, Laura Quinn, Emma Devitt, Giovanni Villa, Sharee A. Basdeo, Liam Townsend

**Affiliations:** 1 School of Medicine, Trinity Translational Medicine Institute, St James’s Hospital, Trinity College Dublin, The University of Dublin, Dublin, Ireland; 2 Department of Genitourinary Medicine and Infectious Diseases, St James’s Hospital, Dublin, Ireland

**Keywords:** antiretroviral therapy, HIV - human immunodeficiency virus, immunometabolism, monocytes, pre-exposure prophylaxis (PrEP), tenofovir disaproxil fumarate and emtricitabine

## Abstract

**Introduction:**

Pre-exposure prophylaxis (PrEP) with tenofovir/emtricitabine (TDF/FTC) is highly effective for HIV prevention. While antiretroviral therapy (ART) is linked to chronic inflammation in people living with HIV, its direct effects on immune phenotype, function, and metabolism in HIV-negative individuals remain unclear. This study aimed to investigate how daily TDF/FTC pre-exposure prophylaxis modulates immune activation, functional responses, and metabolic programming in innate and adaptive immune cells in HIV-negative individuals.

**Methods:**

Gay, bisexual, and other men who have sex with men (gbMSM) on daily TDF/FTC PrEP underwent immunophenotyping and single-cell metabolic profiling using SCENITH™. Cytokine and chemokine responses were measured ex vivo and after lipopolysaccharide or *Mycobacterium tuberculosis* stimulation. Responses were compared with those of a demographically similar PrEP-naïve cohort, and five participants were followed longitudinally for 6–9 months after PrEP initiation.

**Results:**

Monocytes from people taking PrEP (n=15; median 533 days) exhibited higher activation marker expression (HLA-DR, CD14) *ex vivo* and enhanced IL-1β and TNF after bacterial challenge compared with PrEP-naïve individuals (n=11). Longitudinal follow-up of a pilot cohort (n=5) suggested that PrEP initiation increased monocyte activation marker expression (HLA-DR, CD14, CD40, TNFRI/II) and cytokine production (IL-1β, TNF, GM-CSF, IFN-γ, Granzyme B, MIP-1α). Reduced glucose dependency was observed in monocytes, CD56^dim^ NK cells and CD4^+^ T cells 6–9 months after PrEP initiation.

**Discussion:**

Daily TDF/FTC promotes monocyte activation, enhances pro-inflammatory responses, and appears to reprogramme immune cell metabolism, highlighting ART’s potential to modulate immune-mediated inflammatory pathways in HIV-negative individuals.

## Graphical Abstract

**Figure:**
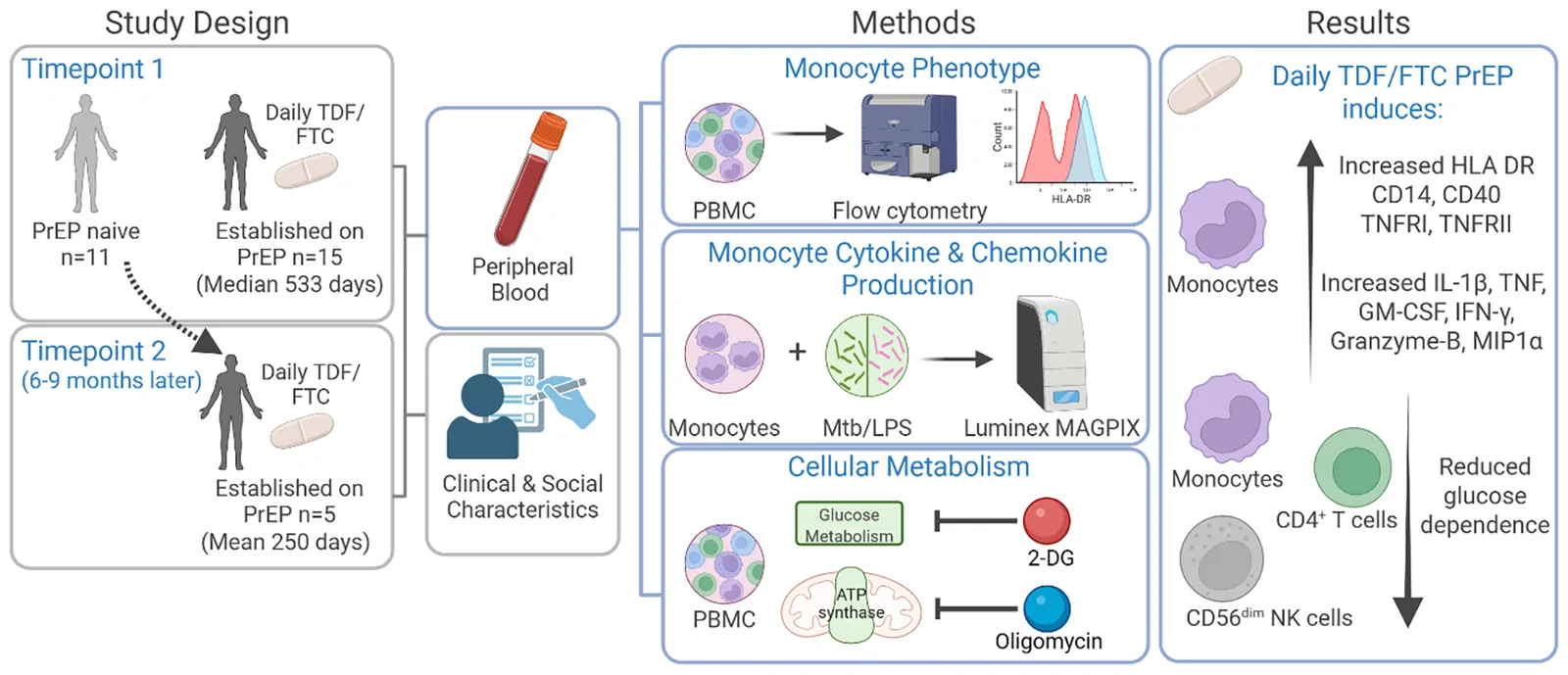
Created in BioRender. Townsend, L. (2026) https://BioRender.com/c0qjf4v.

## Introduction

The evolution of antiretroviral therapies (ART) for human immunodeficiency virus (HIV) infection has transformed the condition from a terminal illness to a chronic lifelong condition (1). The life expectancy of people living with HIV (PLWHIV) on ART is now similar to that of HIV-negative individuals (2). However, this increase in lifespan is not accompanied by a similar increase in healthspan, i.e. years lived in good health. PLWHIV develop ageing-related comorbidities at a younger age than HIV-negative individuals (3, 4). The reasons behind this are multifactorial and incompletely understood. Some of the drivers of this accelerated ageing phenotype are co-infections such as viral hepatitis and lifestyle factors (5, 6). HIV infection remains the strongest risk factor for developing tuberculosis disease (TBD). Importantly, PLWHIV receiving long-term ART and maintaining normal-range CD4 counts remain at increased risk of TBD, and poorer TB treatment outcomes (7–9). Chronic inflammation due to HIV infection, even in the presence of effective anti-retroviral treatment (ART), has also been implicated in the development of ageing-associated comorbidities (10).

ART has been proposed to contribute to the immune dysregulation and altered metabolism that precedes the development of multimorbidity in PLWHIV (11). The role of ART is particularly relevant given its expanded use for HIV prevention, which provides an opportunity to investigate the immunomodulatory effects of ART in the absence of HIV infection. HIV pre-exposure prophylaxis (PrEP), given as a fixed-dose combination of two nucleoside reverse transcriptase inhibitors (NRTIs) tenofovir disoproxil fumarate (TDF) and emtricitabine (FTC), taken either daily or on-demand, is highly effective at preventing HIV infection in individuals at increased risk of HIV acquisition (12, 13). The majority of PrEP users are gay, bisexual and men who have sex with men (gbMSM). There are few studies examining the impact of PrEP on immune responses. Individuals taking PrEP do not develop enduring infection or antibody responses to HIV, despite presumed ongoing exposure to the virus. This suggests innate immune response may remain active in this context and could be modulated by PrEP (14). Additionally, TDF has been demonstrated to alter host cytokine and interferon responses (15, 16).

We hypothesised that PrEP alters innate immune activation and responses to unrelated bacterial stimuli through mechanisms associated with changes in cellular metabolism. We used *Mycobacterium tuberculosis* (Mtb; strain H37Rv) whole cell lysate as a model of complex bacterial stimulation containing multiple agonists for innate cell pathogen recognition receptors (PRRs). This approach also enabled us to explore the effects of ART on innate immune responses to Mtb. In parallel, we used a reductive model of TLR4 only stimulation with LPS, since myeloid challenge with LPS is well defined in animal models and *ex vivo* in healthy humans. Previous *in vitro* studies have shown that exposure to TDF/FTC alters monocyte phenotype and function (17, 18). We compared immune responses in gbMSM taking PrEP to gbMSM with similar lifestyle risk factors who were not taking PrEP. To assess differences in immune responses, we examined monocyte, natural killer (NK) cell and T cell phenotype, function and metabolism in a cohort of gbMSM already established on daily PrEP (n=15) and a cohort of gbMSM prior to commencing PrEP (n=11). We then prospectively followed the cohort commencing PrEP and re-assessed these immune parameters 6–9 months after they were established on daily PrEP (n=5).

## Methods

### Study setting, participants and clinical covariates

Participants were recruited through the PrEP service at St James’s Hospital, Dublin, a large tertiary referral centre. Informed consent was obtained. Eligible participants were cisgender gbMSM aged >18 years, either already on daily PrEP or initiating it. Exclusion criteria included self-reported infection symptoms, detection of asymptomatic sexually transmitted infection (STI) at recruitment, event-based PrEP use, active injection drug use, recent vaccination (within three months), or use of immunomodulatory medication. Sociodemographic data, sexual history, condom use, STI history, including most recent screen undertaken and most recent positive test, substance use, comorbidities, and medications were recorded. All participants underwent STI screening (urine, rectal, and pharyngeal swabs for *Chlamydia trachomatis* and *Neisseria gonorrhoeae*) and serology for HIV, Hepatitis B, Hepatitis C, and *Treponema pallidum*. Blood (40 ml) was collected in lithium heparin tubes. Participants starting daily PrEP were invited for repeat sampling after at least six months, with identical data collected. Adherence to daily PrEP was self-reported in accordance with standard clinical practice, with no therapeutic drug monitoring conducted for this study.

### Peripheral blood mononuclear cell and monocyte isolations

Peripheral blood mononuclear cells (PBMC) were isolated from whole blood samples by density gradient centrifugation over Lymphoprep™ (StemCell Technologies). Monocytes were separated using the EasySep™ Human Monocyte Enrichment Kit without CD16 Depletion (19058; STEMCELL). Monocytes were added to a non-treated 48-well tissue culture plate (200,000 cells/well). Cells were stimulated with *Mycobacterium tuberculosis* (Mtb; strain H37Rv) whole cell lysate (10 μg/ml; BEI Resources) or lipopolysaccharide (LPS; 10 ng/ml; Sigma Aldrich) for 18 hours. Cells were left unstimulated as a control. All assays were performed on freshly isolated cells; no cells were cryopreserved.

### Cytokine and chemokine analyses

A Human Luminex Discovery Kit (Cat LXSAHM-25) was custom designed to assess the following analytes in monocyte supernatants: TNF, IL-10, MCP-1, IL-1β, MIP-1α, IL-17α, GM-CSF, G-CSF, IFN-γ, and Granzyme B. The assay was carried out as per manufacturer’s protocol and analysed on the Luminex MAGPIX system. Unstimulated and stimulated cells from each donor at each timepoint were analysed concurrently on the same plate, thereby eliminating potential inter-assay variability. Analyte concentrations were determined based on standards supplied in the kit with a 5-parameter logistic regression curve used in xPONENT™ software version 4.3. To minimise batch effects across the two time points, all stimulations were prepared prior to study initiation and thawed on the day of use to enhance consistency. To maximise consistency and minimise technical variation, Luminex analyses of T1 and T2 supernatants were performed simultaneously using the same patient samples on the same assay plate.

### Immune cell phenotyping

Phenotypic analyses were carried out using flow cytometry using antibodies purchased from BioLegend. PBMC (100,000/tube) were resuspended in PBS (Gibco) and stained immediately. For lymphocyte phenotyping, cells were stained for 10 minutes at room temperature with anti-CD56 BV421 (1:100; 5.1H11), anti-CD69 BV510 (1:50; FN50), anti-CD8 BV650 (1;100; SK1), anti-CD27 BV785 (1:50;LG.3A10), anti-CD45RO APC-Cy7 (1:33;UCHL1), anti-CXCR6 PerCP-Cy5.5 (1:50; K041E5), anti-CD16 PE-Cy7 (1:200; 3G8), anti-NKG2C PE (1:100; S19005E), anti-CD3 PE-Dazzle (1:100; OKT3), and anti-NKG2A APC (1:100; S19004C).

For monocyte phenotyping, cells were stained with anti-CD86 BV421 (1:100;IT2.2), anti-CD45RA BV711 (1:100; HI100), anti-CD40 APC-Cy7 (1:33; HI100), anti-HLA-DR APC (1:100; L243), anti-CD14 Alexa Fluor 488 (1:100; HCD14), anti-CD16 PerCP-Cy5.5 (1:100; 3G8), anti-CD80 PE-Cy7 (1:100; 2D10), anti-TNFRI PE (1:50; W15099A), and anti-TNFRII PE-Dazzle (1:100; 3G7A02). Cells were washed, fixed and stored at 4 °C until acquisition on the BD LSR Fortessa and analysed using FlowJo™ version 10.1.

### Measuring immune cell metabolism using SCENITH

**Figure 1:**
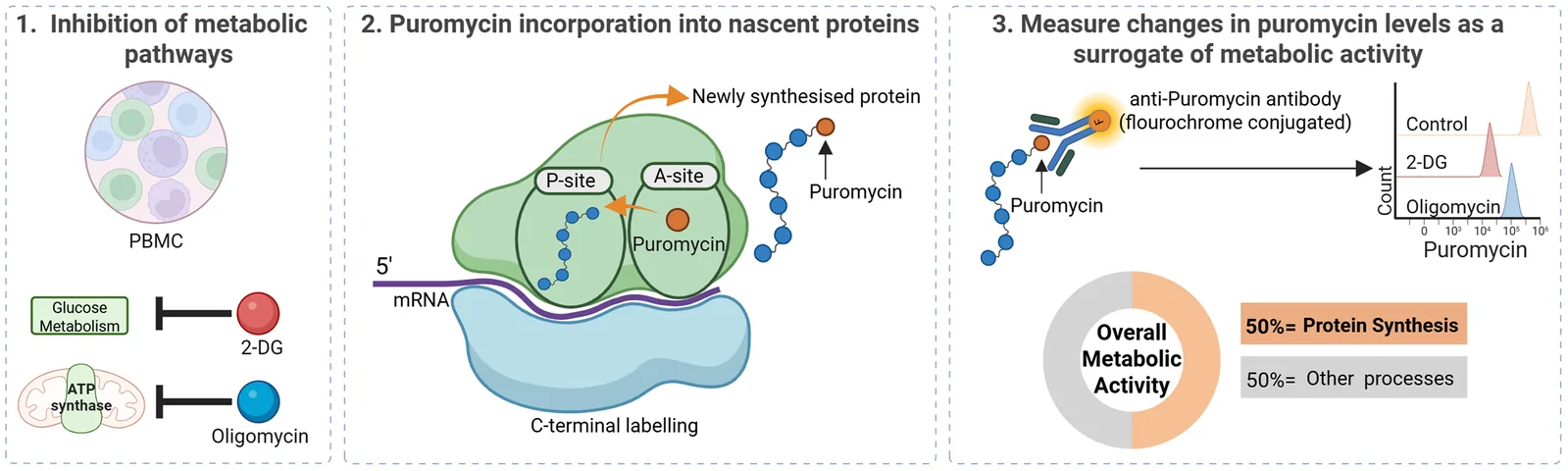
SCENITH protocol overview. Peripheral blood mononuclear cells (PBMC) are treated with metabolic inhibitors targeting specific pathways: 2-deoxyglucose (2-DG) to inhibit glucose metabolism and oligomycin to inhibit mitochondrial ATP synthase. Puromycin, an analogue of aminoacyl-tRNA, is incorporated into nascent polypeptide chains at the ribosomal A-site, resulting in C-terminal labelling of newly synthesized proteins. Incorporated puromycin is then detected by flow cytometry using a fluorochrome-conjugated anti-puromycin antibody, providing a quantitative measure of overall cellular metabolic activity. As protein synthesis accounts for approximately 50% of total metabolic activity, puromycin incorporation serves as a reliable surrogate for assessing cellular metabolic state. Created in BioRender. Connolly, S. (2026) https://BioRender.com/wcxp10k.

The SCENITH protocol (19) uses puromycin as a surrogate for protein synthesis as it becomes incorporated into newly formed proteins (**Figure 1**). SCENITH quantifies relative changes in translation following specific metabolic perturbations, enabling inference of glycolytic and mitochondrial reliance. Cells were treated for 40 mins at 37 °C, 5% CO_2_ with DMSO Control (Co, 0.02% DMSO), 2-DG (100 mM; Sigma-Aldrich), Oligomycin (O; 1 μM; Sigma-Aldrich), a combination of both drugs (DGO), or negative control; Harringtonine (Sigma-Aldrich; 2 µg/ml). Puromycin (Puro; 10 μg/mL; Sigma-Aldrich) was added for the final 35 mins. Cells were washed in PBS and flow cytometry staining was carried out. Cells were blocked with FcR block and stained with Zombie NIR™ viability dye (1:200), anti-CD14 Alexa Fluor488 (1:100; HCD14), anti-CD16 PE-Cy7 (1:200; 3G8), anti-CD56 BV421 (1:100; 5.1H11), anti-CD3 BV510; SK7), anti-CD4 PerCp-Cy5.5 (1:100; A161A1), fixed and permeabilised using eBioscience™ Foxp3/Transcription Factor Staining Buffer Kit (Thermofischer) and intracellularly stained with anti-puromycin (1:100; 2A4; BioLegend) for 30 mins at 4 °C. Cells were acquired on the BD LSR Fortessa and analysed using FlowJo™ version 10.1. Metabolic capacities and dependencies were calculated as described previously (19, 20);

Co = GeoMFI of anti-Puromycin-Fluorochrome upon Control treatment
DG = GeoMFI of anti-Puromycin-Fluorochrome upon 2-Deoxy-D-Glucose treatment
O = GeoMFI of anti-Puromycin-Fluorochrome upon Oligomycin A treatment
DGO = GeoMFI of anti-Puromycin-Fluorochrome upon DG+O treatment
Mitochondrial Dependence (MD)=100(Co-O)/(Co-DGO)
Glucose Dependence (GD)= 100(Co-DG)/(Co-DGO)

### Statistical analyses

Cohort characteristic data was analysed using Stata version 19.0 (Stata Statistical Software). All other statistical analyses were performed using GraphPad Prism 10.4.2 software. The statistical test used is indicated in each figure legend. A P-value of <0.05 was considered significant. Each marker and cytokine was analysed independently; therefore, no correction for multiple comparisons was applied.

## Results

### Participant characteristics

A total of 26 participants were recruited, with n=15 established on daily PrEP and n=11 recruited prior to commencing PrEP (treatment naive). All were gbMSM, with a mean age of 34.4 years. The only comorbidities recorded were asthma, depression and hyperlipidaemia. The only prescribed medications in the cohort were salbutamol, escitalopram and atorvastatin. Eleven participants reported at least one STI in the preceding 12 months; n=8 *N. gonorrhoea*, n=6 *C. trachomatis*, n=1 syphilis and n=1 L*ymphogranuloma venereum*. The demographics between the PrEP and non-PrEP cohorts were similar, with no differences in age, sexual behaviours, or substance misuse (**Table 1**). The median time on TDF/FTC for the cohort taking PrEP was 533 days (IQR 273 – 1,449).

**Table 1:** Cohort characteristics.

	Total cohort (n=26)	Not on PrEP (n=11)	On PrEP (n=15)	Cohort comparison
Time on PrEP, days; median (IQR)	533 (273 – 1,449)		533 (273 – 1,449)	n/a
Age, years; Mean (SD)	34.4 (8.9)	32.9 (11.1)	35.5 (7.0)	*t* = -0.74, p=0.47
Comorbidities				
- None - One - Two	24 (92) 1 (4) 1 (4)	11 (100) 0 (0) 0 (0)	13 (86) 1 (7) 1 (7)	*z=*-1.24, p=0.22
STI in last year, yes; n (%)	11 (42)	4 (36)	7 (47)	χ^2^ = 0.28, p=0.60
Ethnicity; n (%)				
- White Irish - White European - Other White - Asian	9 (35) 6 (23) 10 (38) 1 (4)	3 (27) 3 (27) 4 (36) 1 (9)	6 (40) 3 (20) 6 (40) 0 (0)	r^2^ = 0.03, p=0.39
Partners in last 30 days, n: median (IQR)	3 (2 – 6)	2 (1 – 4)	4 (2 – 6)	z=-1.89, p=0.06
Partners in last 6 months, n: median (IQR)	13.5 (5 – 30)	10 (7 - 27)	24 (6 - 40)	z=-0.96, p=0.35
Condom use; n (%)				
- Always - >50% of time - <50% of time - Never	5 (19) 7 (27) 9 (35) 5 (19)	3 (27) 4 (36) 3 (27) 1 (9)	2 (13) 3 (20) 6 (40) 4 (27)	r^2^ = 0.09, p=0.13
Substance use; n (%)				
- alcohol - cigarettes - cannabis - cocaine - gamma hydroxybutyrate - methamphetamine - MDMA - alkyl nitrites	20 (77) 3 (12) 2 (8) 1 (4) 3 (12) 4 (15) 2 (8) 1 (4)	9 (82) 0 (0) 1 (9) 0 (0) 0 (0) 1 (9) 1 (9) 0 (0)	11 (73) 3 (20) 1 (7) 1 (7) 3 (20) 3 (20) 1 (7) 1 (7)	

Differences assessed using ANOVA, Student’s t test, Wilcoxon rank sum, and Pearson’s chi-square test, as appropriate. PrEP, Pre-exposure prophylaxis; STI, sexually-transmitted infection; MDMA, 3,4-Methylenedioxymethamphetamine.

### PrEP significantly enhanced monocyte proinflammatory phenotype and function

**Figure 2:**
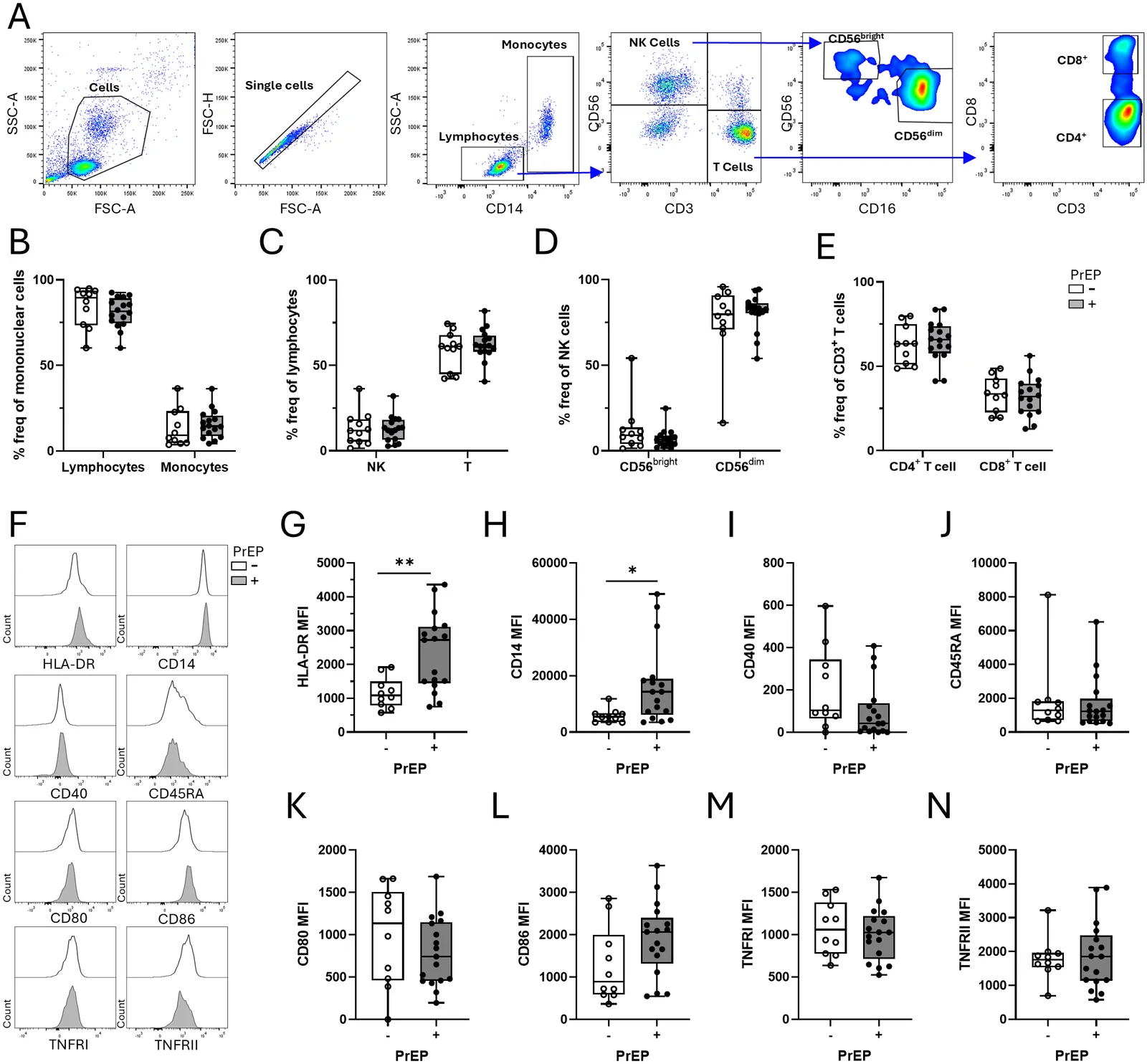
Daily PrEP induces a pro-inflammatory phenotype in peripheral blood monocytes. PBMC were isolated from peripheral blood of PrEP naive individuals (white; n=11) or individuals established on PrEP (grey; n=15) and stained with fluorochrome conjugated antibodies for CD14, CD56, CD3, CD8, HLA-DR, CD40, CD45RA, CD80, CD86, TNFRII and TNFRII for flow cytometric analysis. **(A)** Representative flow plot of gating strategy. **(B)** Lymphocyte and monocyte frequencies as a percentage of total mononuclear cells. **(C)** NK and T cell frequencies as a percentage of total lymphocytes. **(D)** CD56^bright^ and CD56^dim^ frequencies as a percentage of total NK cells. **(E)** CD4^+^ and CD8^+^ frequencies as a percentage of total T cells. **(F)** Representative histograms of the Median Fluorescence Intensity (MFI) of the activation markers on monocytes. Expression levels of **(G)** HLA-DR, **(H)** CD14, **(I)** CD40, **(J)** CD45RA, **(K)** CD80, **(L)** CD86, **(M)** TNFRI or **(N)** TNFRII on total monocytes. Each dot represents an individual donor, and data were analysed using **(B-E)** a two-way ANOVA with Šidák’s multiple comparisons test or **(G-N)** an unpaired t test where *P<0.05, or **P<0.01; **(G)** P = 0.0041, **(H)** P = 0.0245.

Cellular frequencies and phenotype of immune cell subsets present in PBMC from PrEP and non-PrEP participants were measured by flow cytometry. The frequencies of monocytes and lymphocytes were comparable between the two groups (**Figures 2A, B**). Similarly, no significant differences were observed in the frequencies of NK and T cells or their respective subsets, including CD56^bright^, and CD56^dim^ NK cells, and CD4^+^ and CD8^+^ T cells (**Figures 2C–E**). However, PrEP was associated with a pro-inflammatory phenotype in peripheral blood monocytes, characterised by increased expression of the antigen-presentation molecule HLA-DR and the TLR-4 co-receptor CD14 (**Figures 2F–H**). There were no differences observed between groups in monocyte expression of costimulatory molecules CD40, CD45RA, CD80 and CD86, or TNF receptors; TNFRI and TNFRII (**Figures 2I–L**). Extensive phenotyping of NK- and T-cell activation markers was also performed; however, no significant differences were observed (**Supplementary Figures 1A–D**).

**Figure 3:**
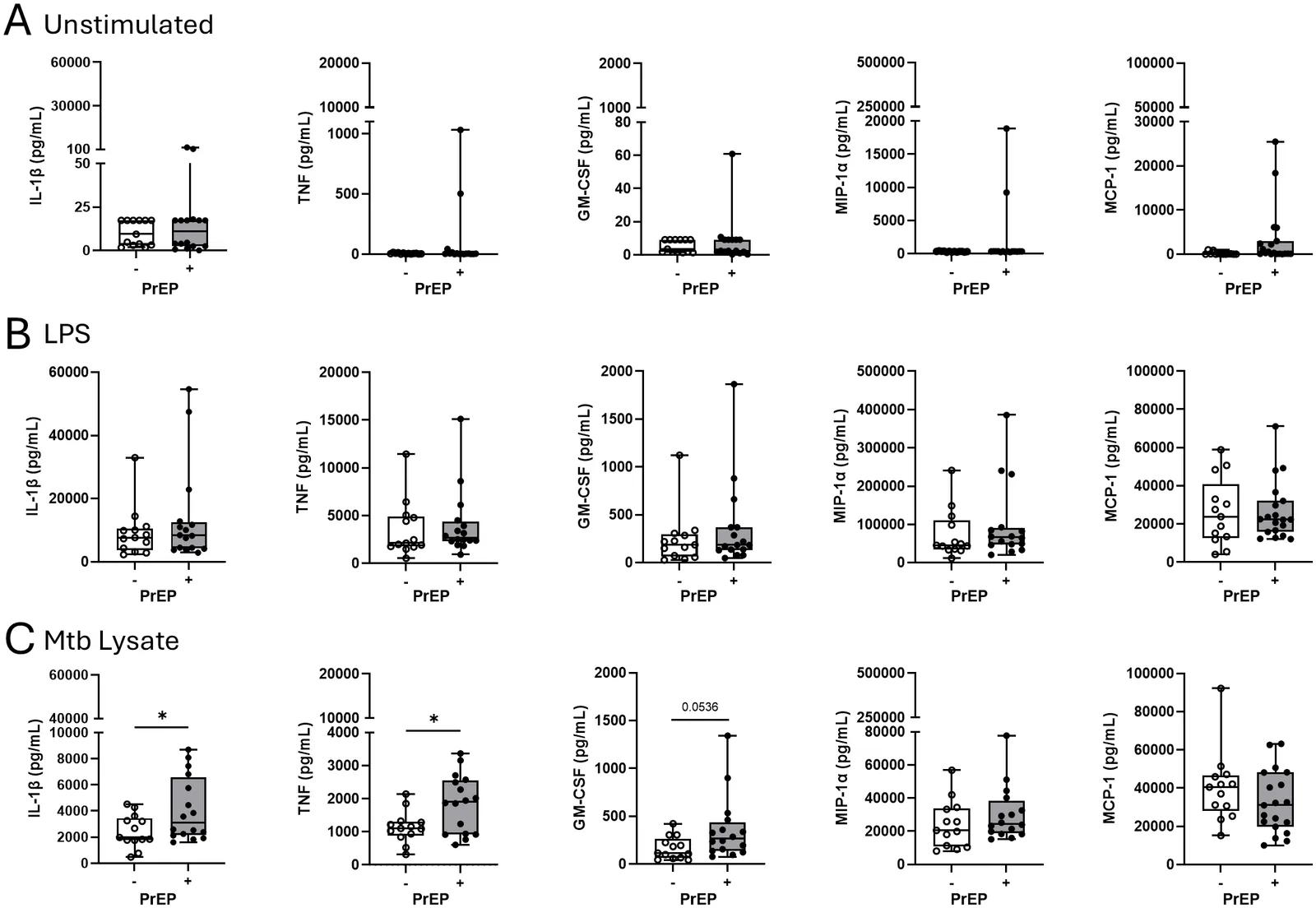
Daily PrEP enhances monocyte pro-inflammatory responses to Mtb lysate. Monocytes were magnetically sorted from the PBMC of PrEP naive individuals (white; n=11) or individuals established on PrEP (grey; n=15) and **(A)** left unstimulated, **(B)** stimulated with LPS (10 ng/ml) or **(C)** stimulated with Mtb lysate (10 μg/ml) for 18 hours. The concentrations (pg/ml) of IL-1β, TNF, GM-CSF, MIP-1α or MCP-1 in the supernatant were measured using a Luminex Discovery assay. Each dot represents an individual donor, and data were analysed using an unpaired t test where *P<0.05. **(C)** IL1-β P = 0.0358, TNF P = 0.0141; both Mann-Whitney.

Given the changes in monocyte markers of activation, monocyte function was next investigated. Monocytes were isolated from PBMC and left unstimulated or stimulated with Mtb lysate (10 μg/ml) or LPS (10 ng/ml) for 18 hours. Production of IL-1β, TNF, GM-CSF, MIP-1α, MCP-1, Granzyme-B, IFN-γ, IL-17, IL-10 and G-CSF were measured under basal conditions (unstimulated), following TLR4 activation by LPS, and following multiple PRR activation with Mtb lysate. Under basal conditions, there were no changes in monocyte cytokine or chemokine production between groups (**Figure 3A**; **Supplementary Figure 2A**). Following stimulation of TLR4 with LPS, increased cytokine and chemokine production was observed. The magnitude of these responses was similar in both the PrEP and non-PrEP groups (**Figure 3B**; **Supplementary Figure 2B**). However, following simultaneous stimulation of multiple PRRs with Mtb lysate, monocytes in the PrEP cohort demonstrated significantly higher production of IL-1β and TNF compared with -PrEP naive participants (**Figure 3C**). GM-CSF, MIP-1α, and MCP-1 were not significantly altered in participants receiving PrEP compared with PrEP-naïve controls (**Figure 3C**). This suggests that daily PrEP results in enhanced production of pro-inflammatory IL-1β and TNF in circulating monocytes. Cytokine and chemokine production following Mtb stimulation, including Granzyme B, IFN-γ, IL-17, IL-10, and G-CSF, was extensively profiled but showed no significant changes (**Supplementary Figure 2C**).

### Longitudinal analysis following treatment initiation indicates that PrEP may increase monocyte pro-inflammatory phenotype and function

Due to the differences in monocyte phenotype and effector function observed between PrEP and non-PrEP participants we next prospectively followed the non-PrEP participants who commenced daily PrEP post timepoint 1. The 11 participants were invited to return for repeat sampling a minimum of six months after PrEP commencement. Six participants were lost from the study; n=2 had switched to event-based dosing, n=3 declined further participation or had disengaged from the service, and n=1 had a positive STI screen at Timepoint 2, leaving n=5 eligible participants. These patients had been taking PrEP for a mean of 250 days (SD 10.8). There were no demographic differences between the eligible n=5 and the six participants who did not provide a timepoint 2 sample.

**Figure 4:**
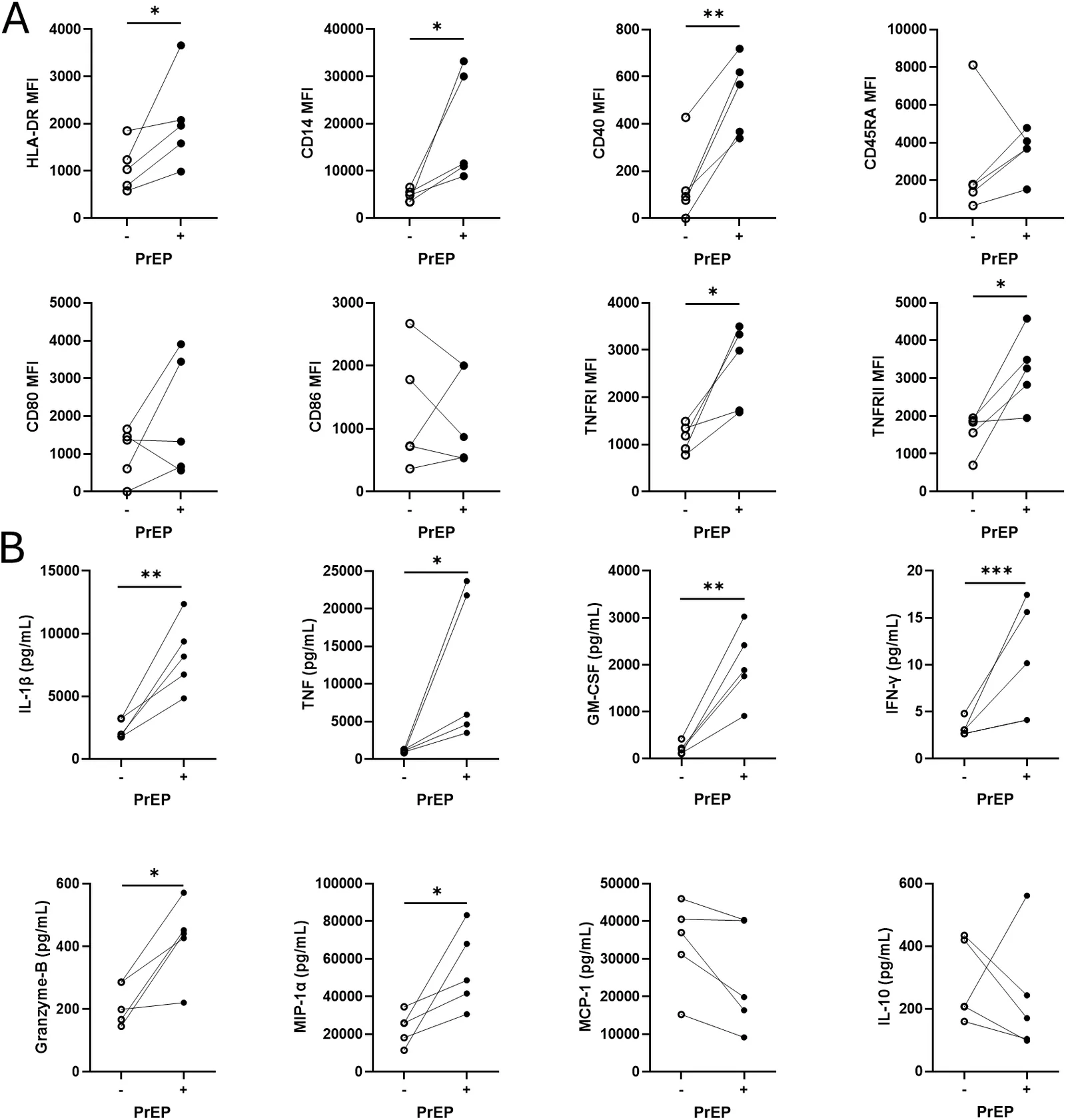
Longitudinal follow up confirms PrEP-treatment effects. PBMC were isolated from the matched blood (n=5) of PrEP naïve individuals at T1 (-; open circle) who were established on PrEP at T2 (+; closed circle) and stained with fluorochrome conjugated antibodies for CD14, HLA-DR, CD40, CD45RA, CD80, CD86, TNFRII and TNFRII for flow cytometric analysis. **(A)** Expression levels of HLA-DR, CD14, CD40, CD45RA, CD80, CD86, TNFRI or TNFRII on total monocytes. **(B)** Magnetically-sorted monocytes were stimulated with Mtb lysate (10 μg/ml) for 18 hours. The concentrations (pg/ml) of IL-1β, TNF, GM-CSF, IFN-γ, Granzyme-B, MIP-1α, MCP-1, and IL-10 in the supernatant were measured using a Luminex Discovery assay. Each dot represents matched data at each timepoint joined by a line. Data were analysed using a paired t test where *P<0.05, **P<0.01, or ***P<0.001. **(A)** HLA-DR P = 0.0326, CD14 P = 0.0265 (both one-tailed paired t test), CD40 P = 0.0028, TNFRI P = 0.0202, TNFRII P = 0.0258 (all two-tailed). **(B)** IL-1β P = 0.0072, GM-CSF P = 0.0048, IFN-γ P = 0.0004, Granzyme B P = 0.0193, MIP-1α P = 0.0412 (all two-tailed paired t test), TNF P = 0.0363 (one-tailed paired t test).

Once established on daily PrEP, markers of monocyte activation, namely HLA-DR, CD14, CD40, TNFRI and TNFRII expression levels were significantly higher compared with timepoint 1 (**Figure 4A**; gating strategy and representative histograms in **Figures 2A, F**). Following Mtb stimulation (Mtb lysate 10 μg/ml for 18 h), there were marked increases across a range of cytokines produced by monocytes, with significantly higher concentrations of IL-1β, TNF, GM-CSF, IFN-γ, Granzyme-B, and MIP-1α, produced compared with their own matched PrEP-naive control from timepoint 1 (**Figure 4B**; **Supplementary Figure 3C**). Monocyte IFN-γ and MCP-1 production were significantly increased post initiation of PrEP under basal conditions, and IL-1β and GM-CSF were significantly increased after stimulation with LPS (10 ng/ml for 18 h; **Supplementary Figures 3A, B**).

### PrEP metabolically reprogrammes myeloid and lymphoid cell compartments

**Figure 5:**
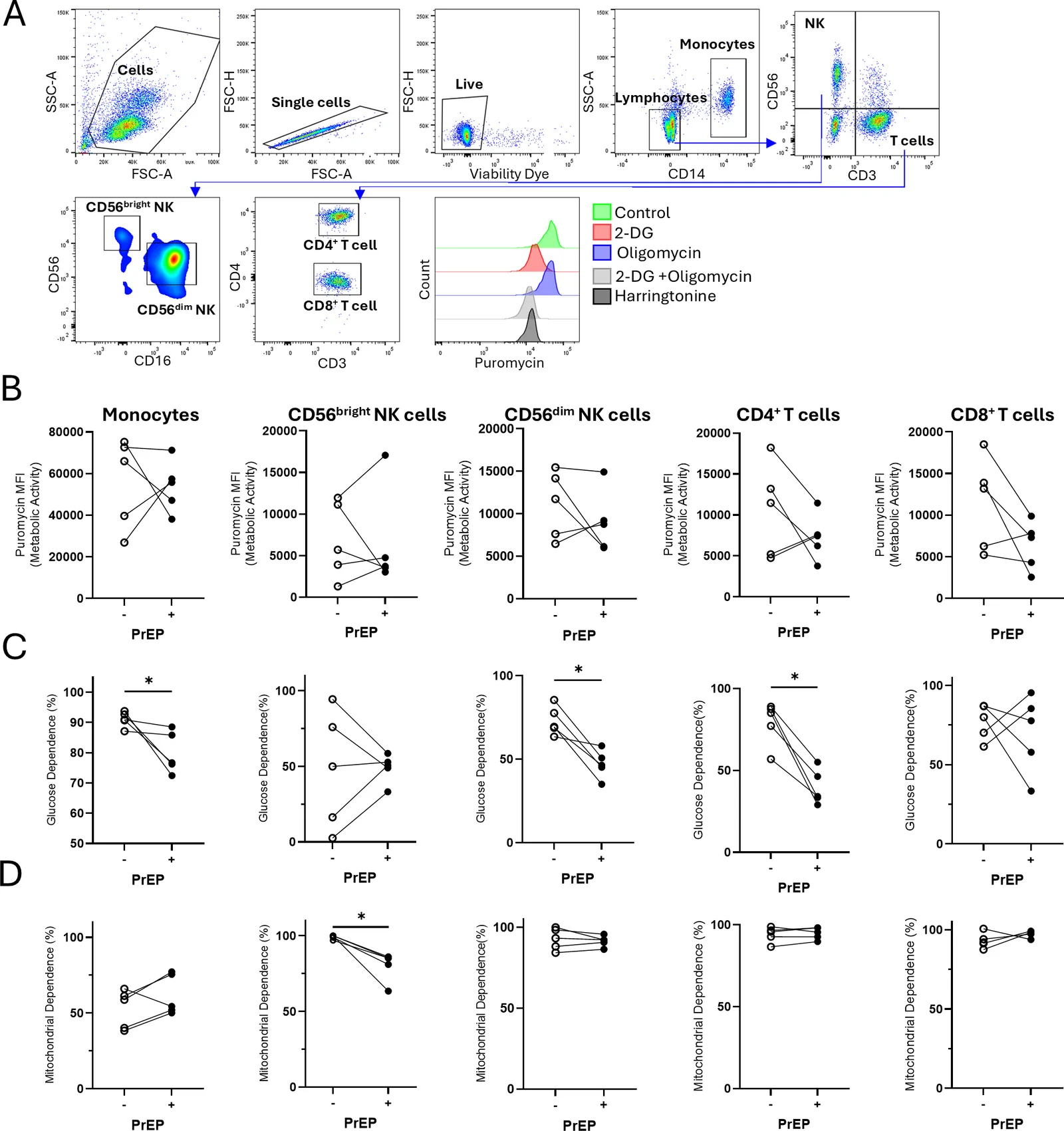
Daily PrEP induces metabolic reprogramming in myeloid and lymphoid cells. PBMC from (n=5) individuals who were PrEP naïve at T1 (-; open circle) and on PrEP on T2 (+; closed circle) were treated with DMSO control or metabolic inhibitors 2-deoxyglucose (2DG), oligomycin, or combinations of both, or harringtonine, which inhibits protein synthesis and acts as a negative control for this assay. Puromycin was then added, and cells were cultured for 40 min. Cells were washed and stained with fluorochrome-conjugated antibodies against CD14, CD56, CD3, CD4 and puromycin and analysed by flow cytometry. Analysis was performed on monocytes, CD56^bright^ NK cells, CD56^dim^ NK cells, CD4^+^ T cells and CD8^+^ T cells **(A)** Representative flow plot of gating strategy showing the analyses of monocytes and their puromycin staining across metabolic treatments. **(B)** Puromycin MFI values measuring overall metabolic activity in cells *ex vivo* across Ctrl (DMSO control), 2DG, and oligomycin treatments. **(C)** Percentage glucose dependence in cells *ex vivo*. **(D)** Percentage mitochondrial dependence in cells *ex vivo*. Each dot represents an individual donor and matched data at each timepoint joined by a line. Data were analysed using a paired t test where *P<0.05. **(C)** Glucose Dependence monocytes P = 0.0489 (two-tailed paired t test), CD56^dim^NK cells P = 0.0312 and CD4^+^T cells P = 0.0312 (both Wilcoxon matched pairs one-tailed). **(D)** Mitochondrial Dependence CD56^bright^ NK cells P = 0.0135 (two-tailed paired t test).

Since innate immune function is integrally linked to cellular metabolism, we hypothesised that the observed phenotypic and functional changes in monocytes from people established on PrEP may be a result of a PrEP-induced metabolic reprogramming. We therefore investigated whether PrEP alters monocyte metabolic profiles using the SCENITH flow cytometry-based metabolic profiling platform (**Figure 1**) (19). We took advantage of this protocol’s ability to assess metabolic profiles at a single cell resolution and also analysed CD56^bright^ NK cells, CD56^dim^ NK cells, CD4^+^ T cells and CD8^+^ T cells. Using puromycin incorporation as a surrogate marker of translation, which accounts for approximately 50% of cellular metabolic activity, we assessed overall cellular metabolism in PrEP-naïve individuals and again 6–9 months after PrEP initiation. Basal puromycin incorporation was comparable between groups, indicating that global translational activity was not suppressed and that there were no significant changes in overall metabolic activity between timepoints (**Figures 5A, B**). To evaluate glucose dependency, cells were treated with the glucose analogue 2-deoxyglucose (2-DG) to inhibit glucose metabolism. PrEP initiation resulted in a reduction in glucose dependency in monocytes, CD56^dim^ NK cells and CD4^+^ T cells, but remained unchanged in CD56^bright^ NK cells and CD8^+^ T cells (**Figure 5C**). Inhibition of mitochondrial respiration using oligomycin enabled assessment of mitochondrial dependency. These analyses revealed that PrEP establishment reduced mitochondrial dependency in CD56^bright^ NK cells while no significant changes were observed in monocytes, CD56^dim^ NK, CD4^+^, or CD8^+^ T cells (**Figure 5D**).

Taken together, these data indicate that initiation of daily PrEP is associated with altered innate immune phenotype and function and changes in the metabolism of circulating myeloid and lymphoid cells.

## Discussion

The contribution of HIV ART, particularly TDF and FTC, to altered inflammatory responses remains poorly understood. Here, we demonstrate that daily TDF/FTC PrEP induces measurable changes in immune cell phenotype, function, and metabolism in otherwise healthy gbMSM, independent of HIV infection. A nested longitudinal pilot study following five individuals from PrEP initiation through more than six months of daily use suggests that these changes are dynamic and may relate to duration of PrEP exposure, providing important preliminary data to establish larger prospective cohort studies. Our findings indicate that PrEP not only exerts antiviral effects but may also modulate innate and adaptive immune responses *in vivo*, supporting previous *in vitro* studies that have highlighted the immunomodulatory properties of TDF/FTC (16, 21).

Monocytes from PrEP users exhibited an activated, pro-inflammatory phenotype, with increased expression of HLA-DR, CD14, CD40, TNFRI, and TNFRII. In the cross-sectional comparison (**Figure 1**), increased expression was observed only for HLA-DR and CD14. These changes were more pronounced in the small longitudinal cohort followed from PrEP initiation to established daily use, with additional increases in CD40, TNFRI, and TNFRII (**Figure 5**). This pattern may indicate that the effects of PrEP exposure on monocyte phenotype may evolve over time, suggesting a temporal component to monocyte activation as well as possible inter-individual variability. Validation of these findings in a larger cohort is required to confirm these results and account for potential confounders. HLA-DR upregulation suggests enhanced antigen presentation capacity, while CD14 and CD40 indicate priming for microbial sensing and T cell activation. Elevated CD40 has also been linked to monocyte adhesion and cardiovascular risk (22), while increased TNFRI and TNFRII expression suggests enhanced TNF-mediated responsiveness while maintaining regulatory capacity (23). Together, these findings provide insight into the impact of TDF/FTC on inducing a pro-inflammatory state in the peripheral myeloid compartment, consistent with observations of elevated plasma markers of monocyte activation in PLWHIV on ART (24, 25). Our longitudinal analysis further suggests that early adaptive changes in cytokine receptor expression may occur during initial PrEP exposure, underscoring the need for larger, longer-term studies to better capture inter-individual variability in monocyte phenotype, function, and metabolism.

Functionally, PrEP monocytes produced higher levels of IL-1β, TNF, GM-CSF, IFN-γ, Granzyme-B, and MIP-1α following Mtb lysate stimulation. While IL-1β and TNF promote host defence, excessive production may contribute to immunopathology (26). GM-CSF stimulates myeloid cell proliferation and enhances neutrophil recruitment, and IFN-γ activates macrophages to improve intracellular pathogen killing. Granzyme-B mediates cytotoxicity against infected cells, including Mtb-infected targets, whereas MIP-1α recruits monocytes and other immune cells to sites of infection, propagating inflammation (27, 28). These findings are in line with prior studies showing that nucleoside reverse transcriptase inhibitors can influence monocyte cytokine production and activation marker expression (18).

Basal cytokine levels in unstimulated monocytes remained largely unchanged, suggesting that PrEP may prime immune cells without inducing overt systemic inflammation under resting conditions. This selective enhancement of responsiveness may be beneficial for host defence, particularly against opportunistic infections such as Mtb, although elevated levels of pro-inflammatory cytokines, especially TNF and IL-1β, have been linked to chronic inflammation, accelerated cardiovascular disease, and worse outcomes in acute infections (29–31). TB remains the leading cause of death in PLWHIV globally, and initiation of ART in TB patients can trigger TB-associated immune reconstitution inflammatory syndrome (TB-IRIS). Transcriptomic analyses reveal that innate immune mediators, including TLR and IL-1 pathways, are strongly upregulated at the characteristic time of TB-IRIS onset (approximately 2 weeks post ART initiation) (32, 33). Consistently, in our study, cytokine responses to LPS were stable, whereas Mtb lysate elicited markedly enhanced cytokine and chemokine production post-PrEP initiation. These data suggest that PrEP may selectively prime monocytes for a more robust pro-inflammatory response to complex pathogen-associated molecular patterns (PAMPs), potentially enhancing host defence, but also raising the possibility of maladaptive inflammation in certain contexts. Notably, in the longitudinal pilot cohort (n=5), MCP-1, a critical signal driving atherosclerosis, was significantly elevated in unstimulated monocytes post-PrEP initiation compared with their own pre-treatment control.

Metabolic profiling using SCENITH revealed that PrEP initiation is associated with reduced glucose dependency in monocytes, CD56^dim^ NK cells, and CD4^+^ T cells, as well as reduced mitochondrial dependency in CD56^bright^ NK cells. This reduced glucose dependency likely reflects lower basal glycolytic activity, which may indicate an enhanced capacity to upregulate glycolysis in response to infection (34, 35). However, older NRTIs have well-recognised mitochondrial toxicities (36, 37). TDF has been known to inhibit DNA polymerases, including mitochondrial DNA polymerase-γ (38, 39). Previous *ex vivo* work has demonstrated that TDF/FTC can modulate monocyte-derived macrophage metabolism, with reduced mitochondrial mass and increased reactive oxygen species production (40). Consequently, the observed reduction in glucose reliance among unstimulated cells may represent a compensatory response to TDF-induced mitochondrial toxicity. This may reflect impaired innate immunity or broader metabolic reprogramming induced by PrEP. Further studies are needed to evaluate the metabolic flexibility of these immune cells following challenge, as well as the effects on immune cells in other anatomical sites, e.g. lungs (41). These findings indicate that TDF/FTC induces metabolic reprogramming across both myeloid and lymphoid compartments. Future studies incorporating complementary metabolic measurements will help further define the mechanisms underlying the metabolic changes observed in PrEP users.

In the context of our study, these TDF/FTC-induced metabolic changes, together with a pro-inflammatory monocyte phenotype, may contribute to the low-grade chronic inflammation observed in PLWHIV. At the same time, our findings suggest that PrEP may enhance metabolic flexibility, potentially enabling immune cells to mount more robust responses to environmental stress or infection. Whether these metabolic shifts confer altered immune surveillance, enhanced protection against pathogens, increased risk of maladaptive inflammation, or are a compensatory response to upstream drug-induced toxicity, remains to be determined.

Our study has limitations. Inflammation in PLWHIV is influenced by complex factors, including diverse social backgrounds, comorbidities, prior ART exposure, and health behaviours. We mitigated many confounders by carefully matching PrEP and non-PrEP cohorts, who shared lifestyle risk factors (42, 43), and all participants had only received TDF/FTC. The mean age of 34.4 years reduces potential effects of immunosenescence and the senescence-associated secretory phenotype (44). PrEP adherence was self-reported and therapeutic drug monitoring was not performed. Due to study attrition, only five participants met eligibility criteria for the longitudinal arm. The findings from these participants provide important preliminary data and a foundation for larger prospective studies specifically aimed at assessing changes in monocyte function and metabolism across the patient’s PrEP journey.

The cohort included only healthy gbMSM, limiting generalizability to women, transgender individuals, or those with comorbidities. Larger studies, including women and transgender patients taking hormonal therapies and powered to determine sex-specific effects of ART on immunity, are therefore warranted. This is particularly important, given the recognised effects of sex hormones on monocyte function and immune metabolism (45–47). Our cohorts were well-matched in terms of number of sexual partners, STI diagnoses in the preceding year, and substance misuse, with no intravenous drug use reported across the cohort. However, this study was not powered to detect differences across specific sexual behaviours or specific recreational drugs and may cause confounding of our findings. Furthermore, stool samples or rectal swabs were not obtained, so the influence of PrEP on the gut microbiome and its relationship with our circulating immune cell findings were not investigated. Only daily TDF/FTC was assessed, so effects of event-based dosing or alternative regimens such as tenofovir alafenamide/emtricitabine or lenacapavir remain unknown. We did not assess viral or HIV peptide responses *ex vivo*, nor cytomegalovirus serostatus, although matched longitudinal sampling partially mitigates this. The study was also not powered to detect clinical events from altered inflammation. These findings support the need for longitudinal PrEP registries to assess long-term outcomes and refine risk–benefit evaluations.

We demonstrate that daily TDF/FTC PrEP shifts inflammatory responses, with some changes persisting over time. Comparing individuals with shorter and longer durations of PrEP exposure suggested sustained increases in CD14 and HLA-DR on monocytes and elevated IL-1β and TNF production, whereas CD40 was only transiently elevated at earlier timepoints. These results suggest that PrEP’s effects on monocyte phenotype and function evolve over time, but whether these changes result in any clinically apparent benefit or harm remains to be seen. This highlights the need for longitudinal studies to identify sub-cohorts at risk of pathological inflammation and to identify clinically relevant consequences of altered monocyte activation.

In summary, daily TDF/FTC PrEP is associated with a pro-inflammatory monocyte phenotype, heightened cytokine responses, and metabolic reprogramming across immune cells, revealing previously unrecognized off-target immunomodulatory effects. While the long-term clinical impact remains unclear, PrEP may enhance host defence but also contribute to low-grade inflammation. Time-dependent changes, such as in CD40, highlight dynamic immune modulation. Longitudinal studies in diverse populations are needed to assess the durability, functional consequences, and clinical relevance of these shifts, informing the long-term risk–benefit profile of PrEP.

## Data Availability

The original contributions presented in the study are included in the article/**Supplementary Material**. Further inquiries can be directed to the corresponding author.

## References

[1] Tseng A Seet J Phillips EJ . The evolution of three decades of antiretroviral therapy: challenges, triumphs and the promise of the future. Br J Clin Pharmacol. (2015) 79:182–94. doi: 10.1111/bcp.12403 24730660 PMC4309625

[2] Trickey A Sabin CA Burkholder G Crane H d'Arminio Monforte A Egger M . Life expectancy after 2015 of adults with HIV on long-term antiretroviral therapy in Europe and North America: a collaborative analysis of cohort studies. Lancet HIV. (2023) 10:e295–307. doi: 10.1016/s2352-3018(23)00028-0 36958365 PMC10288029

[3] Nanditha NGA Paiero A Tafessu HM St-Jean M McLinden T Justice AC . Excess burden of age-associated comorbidities among people living with HIV in British Columbia, Canada: a population-based cohort study. BMJ Open. (2021) 11:e041734. doi: 10.1136/bmjopen-2020-041734 33419911 PMC7799128

[4] Collins LF Palella FJ Jr. Mehta CC Holloway J Stosor V Lake JE . Aging-related comorbidity burden among women and men with or at-risk for HIV in the US, 2008-2019. JAMA Netw Open. (2023) 6:e2327584. doi: 10.1001/jamanetworkopen.2023.27584 37548977 PMC10407688

[5] Prakoeswa FRS Maharani F Bestari RS Aisyah R Ichsan B Nursanto D . Aging and HIV: recent findings in contributing factors. AIDS Res Treat. (2025) 2025:8814760. doi: 10.1155/arat/8814760 40255985 PMC12008487

[6] Helleberg M Afzal S Kronborg G Larsen CS Pedersen G Pedersen C . Mortality attributable to smoking among HIV-1-infected individuals: a nationwide, population-based cohort study. Clin Infect Dis. (2013) 56:727–34. doi: 10.1093/cid/cis933 23254417

[7] Gupta RK Rice B Brown A Thomas L Zenner D Anderson L . Does antiretroviral therapy reduce HIV-associated tuberculosis incidence to background rates? A national observational cohort study from England, Wales, and Northern Ireland. Lancet HIV. (2015) 2:e243–51. doi: 10.1016/s2352-3018(15)00063-6 26423197

[8] Tornheim JA Dooley KE . Tuberculosis associated with HIV infection. Microbiol Spectr. (2017) 5:Ch34. doi: 10.1128/9781555819866.ch34 PMC11687440 28233512

[9] Baniasad A Chao S Nguyen JA Tian E Modongo C Minin VM . HIV remains a risk factor for unfavorable tuberculosis treatment outcomes in the era of universal access to antiretroviral therapy in Botswana. In: Medrxiv (2025). doi: 10.1101/2025.11.26.25340699

[10] Deeks SG Tracy R Douek DC . Systemic effects of inflammation on health during chronic HIV infection. Immunity. (2013) 39:633–45. doi: 10.1016/j.immuni.2013.10.001 24138880 PMC4012895

[11] Erlandson KM Allshouse AA Jankowski CM Lee EJ Rufner KM Palmer BE . Association of functional impairment with inflammation and immune activation in HIV type 1-infected adults receiving effective antiretroviral therapy. J Infect Dis. (2013) 208:249–59. doi: 10.1093/infdis/jit147 23559466 PMC3685225

[12] Molina JM Charreau I Spire B Cotte L Chas J Capitant C . Efficacy, safety, and effect on sexual behaviour of on-demand pre-exposure prophylaxis for HIV in men who have sex with men: an observational cohort study. Lancet HIV. (2017) 4:e402–10. doi: 10.1016/s2352-3018(17)30089-9 28747274

[13] Molina JM Capitant C Spire B Pialoux G Cotte L Charreau I . On-demand preexposure prophylaxis in men at high risk for HIV-1 infection. N Engl J Med. (2015) 373:2237–46. doi: 10.1056/nejmoa1506273 26624850

[14] Hooftman A Peace CG Ryan DG Day EA Yang M McGettrick AF . Macrophage fumarate hydratase restrains mtRNA-mediated interferon production. Nature. (2023) 615:490–8. doi: 10.1038/s41586-023-05720-6 36890227 PMC10411300

[15] Murata K Tsukuda S Suizu F Kimura A Sugiyama M Watashi K . Immunomodulatory mechanism of acyclic nucleoside phosphates in treatment of hepatitis B virus infection. Hepatology. (2020) 71:1533–45. doi: 10.1002/hep.30956 31529730

[16] Melchjorsen J Risor MW Sogaard OS O'Loughlin KL Chow S Paludan SR . Tenofovir selectively regulates production of inflammatory cytokines and shifts the IL-12/IL-10 balance in human primary cells. J Acquir Immune Defic Syndr. (2011) 57:265–75. doi: 10.1097/qai.0b013e3182185276 21471820

[17] Wallace J Gonzalez H Rajan R Narasipura SD Virdi AK Olali AZ . Anti-HIV drugs cause mitochondrial dysfunction in monocyte-derived macrophages. Antimicrob Agents Chemother. (2022) 66:e0194121. doi: 10.1128/aac.01941-21 35293780 PMC9017340

[18] Bowman ER Cameron C Richardson B Kulkarni M Gabriel J Kettelhut A . *In vitro* exposure of leukocytes to HIV preexposure prophylaxis decreases mitochondrial function and alters gene expression profiles. Antimicrob Agents Chemother. (2020) 65(1):e01755-20. doi: 10.1128/aac.01755-20 33020165 PMC7927818

[19] Arguello RJ Combes AJ Char R Gigan JP Baaziz AI Bousiquot E . SCENITH: a flow cytometry-based method to functionally profile energy metabolism with single-cell resolution. Cell Metab. (2020) 32:1063–75:e7. doi: 10.1016/j.cmet.2020.11.007 PMC8407169 33264598

[20] Jameson G Walsh A Woods R Batten I Murphy DM Connolly SA . Human tissue-resident NK cells in the lung have a higher glycolytic capacity than non-tissue-resident NK cells in the lung and blood. Proc Natl Acad Sci USA. (2024) 121:e2412489121. doi: 10.1073/pnas.2412489121 39378091 PMC11494342

[21] Murata K Asano M Matsumoto A Sugiyama M Nishida N Tanaka E . Induction of IFN-lambda3 as an additional effect of nucleotide, not nucleoside, analogues: a new potential target for HBV infection. Gut. (2018) 67:362–71. doi: 10.1136/gutjnl-2016-312653 27789659 PMC5868296

[22] Lont S Mohr F Hecker M Wagner AH . Role of CD40 ligand-mediated endothelial cell-monocyte interaction at atherosclerosis predilection sites. Biochem Pharmacol. (2022) 206:115298. doi: 10.1016/j.bcp.2022.115298 36243097

[23] Gibbings SL Haist KC Redente EF Henson PM Bratton DL . TNFalpha: TNFR1 signaling inhibits maturation and maintains the pro-inflammatory programming of monocyte-derived macrophages in murine chronic granulomatous disease. Front Immunol. (2024) 15:1354836. doi: 10.3389/fimmu.2024.1354836 38404573 PMC10884288

[24] Booiman T Wit FW Maurer I De Francesco D Sabin CA Harskamp AM . High cellular monocyte activation in people living with human immunodeficiency virus on combination antiretroviral therapy and lifestyle-matched controls is associated with greater inflammation in cerebrospinal fluid. Open Forum Infect Dis. (2017) 4:ofx108. doi: 10.1093/ofid/ofx108 28680905 PMC5494939

[25] Robertson J Eden A Yilmaz A Andersson LM Hagberg L Nystrom K . Increased immune activation in people living with HIV on antiretroviral therapy but not when compared with persons on HIV preexposure prophylaxis. Infect Dis (Lond). (2025) 57:1028–35. doi: 10.1080/23744235.2025.2515157 40497636

[26] Saperstein S Chen L Oakes D Pryhuber G Finkelstein J . IL-1beta augments TNF-alpha-mediated inflammatory responses from lung epithelial cells. J Interferon Cytokine Res. (2009) 29:273–84. doi: 10.1089/jir.2008.0076 19231998 PMC2718541

[27] Mattila JT Maiello P Sun T Via LE Flynn JL . Granzyme B-expressing neutrophils correlate with bacterial load in granulomas from Mycobacterium tuberculosis-infected cynomolgus macaques. Cell Microbiol. (2015) 17:1085–97. doi: 10.1111/cmi.12428 PMC4570831 25653138

[28] Arbués A SS Reinhard M Borrell S Gagneux S Portevin D . Soluble immune mediators orchestrate protective *in vitro* granulomatous responses across Mycobacterium tuberculosis complex lineages. eLife. (2025) 13:RP99062. doi: 10.7554/eLife.99062 40162896 PMC11957536

[29] Ridker PM Everett BM Thuren T MacFadyen JG Chang WH Ballantyne C . Antiinflammatory therapy with canakinumab for atherosclerotic disease. N Engl J Med. (2017) 377:1119–31. doi: 10.1056/nejmoa1707914 28845751

[30] Townsend L Dyer AH Naughton A Imangaliyev S Dunne J Kiersey R . Severe COVID-19 is characterised by inflammation and immature myeloid cells early in disease progression. Heliyon. (2022) 8:e09230. doi: 10.1016/j.heliyon.2022.e09230 35386227 PMC8973020

[31] Popa C Netea MG van Riel PL van der Meer JW Stalenhoef AF . The role of TNF-alpha in chronic inflammatory conditions, intermediary metabolism, and cardiovascular risk. J Lipid Res. (2007) 48:751–62. doi: 10.1194/jlr.r600021-jlr200 17202130

[32] Sossen B Kubjane M Meintjes G . Tuberculosis and HIV coinfection: progress and challenges towards reducing incidence and mortality. Int J Infect Dis. (2025) 155:107876. doi: 10.1016/j.ijid.2025.107876 40064284 PMC12957463

[33] Lanzafame M Vento S . Tuberculosis-immune reconstitution inflammatory syndrome. J Clin Tuberc Other Mycobact Dis. (2016) 3:6–9. doi: 10.1016/j.jctube.2016.03.002 31723680 PMC6850228

[34] Gleeson LE Sheedy FJ Palsson-McDermott EM Triglia D O'Leary SM O'Sullivan MP . Cutting edge: Mycobacterium tuberculosis induces aerobic glycolysis in human alveolar macrophages that is required for control of intracellular bacillary replication. J Immunol. (2016) 196:2444–9. doi: 10.4049/jimmunol.1501612 26873991

[35] Cumming BM Addicott KW Adamson JH Steyn AJ . Mycobacterium tuberculosis induces decelerated bioenergetic metabolism in human macrophages. Elife. (2018) 7:e39169. doi: 10.7554/elife.39169 30444490 PMC6286123

[36] Kakuda TN . Pharmacology of nucleoside and nucleotide reverse transcriptase inhibitor-induced mitochondrial toxicity. Clin Ther. (2000) 22:685–708. doi: 10.1016/s0149-2918(00)90004-3 10929917

[37] McComsey GA Walker UA . Role of mitochondria in HIV lipoatrophy: insight into pathogenesis and potential therapies. Mitochondrion. (2004) 4:111–8. doi: 10.1016/j.mito.2004.05.008 16120376

[38] Birkus G Hajek M Kramata P Votruba I Holy A Otova B . Tenofovir diphosphate is a poor substrate and a weak inhibitor of rat DNA polymerases alpha, delta, and epsilon*. Antimicrob Agents Chemother. (2002) 46(5):1610–3. doi: 10.1128/AAC.46.5.1610-1613.2002 PMC127178 11959615

[39] Lewis W Copeland WC Day BJ . Mitochondrial DNA depletion, oxidative stress, and mutation: mechanisms of dysfunction from nucleoside reverse transcriptase inhibitors. Lab Invest. (2001) 81:777–90. doi: 10.1038/labinvest.3780288 11406640

[40] Johnson AA Ray AS Hanes J Suo Z Colacino JM Anderson KS . Toxicity of antiviral nucleoside analogs and the human mitochondrial DNA polymerase. J Biol Chem. (2001) 276:40847–57. doi: 10.1074/jbc.m106743200 11526116

[41] Cox DJ Connolly SA Ó Maoldomhnaigh C Brugman AAI Sandby Thomas O Duffin E . Human airway macrophages are metabolically reprogrammed by IFN-γ resulting in glycolysis-dependent functional plasticity. Elife. (2024) 13:RP98449. doi: 10.7554/elife.98449 39620891 PMC11611294

[42] Ogbuagu O Marshall BDL Tiberio P Ogunbajo A Barakat L Montgomery M . Prevalence and correlates of unhealthy alcohol and drug use among men who have sex with men prescribed HIV pre-exposure prophylaxis in real-world clinical settings. AIDS Behav. (2019) 23:190–200. doi: 10.1007/s10461-018-2260-9 30145707 PMC7020905

[43] Traeger MW Cornelisse VJ Asselin J Price B Roth NJ Willcox J . Association of HIV preexposure prophylaxis with incidence of sexually transmitted infections among individuals at high risk of HIV infection. JAMA. (2019) 321:1380–90. doi: 10.1001/jama.2019.2947 30964528 PMC6459111

[44] Wang B Han J Elisseeff JH Demaria M . The senescence-associated secretory phenotype and its physiological and pathological implications. Nat Rev Mol Cell Biol. (2024) 25:958–78. doi: 10.1038/s41580-024-00727-x 38654098

[45] Taneja V . Sex hormones determine immune response. Front Immunol. (2018) 9:1931. doi: 10.3389/fimmu.2018.01931 30210492 PMC6119719

[46] Hoffmann JP Liu JA Seddu K Klein SL . Sex hormone signaling and regulation of immune function. Immunity. (2023) 56:2472–91. doi: 10.1016/j.immuni.2023.10.008 37967530

[47] De Maeyer RPH Sikora J Bracken OV Shih B Lloyd AF Peckham H . Age-associated inflammatory monocytes are increased in menopausal females and reversed by hormone replacement therapy. Aging Cell. (2025) 24:e70249. doi: 10.1101/2025.03.28.645904 41064932 PMC12611317

