# Supplementary material for: Daily HIV pre-exposure prophylaxis enhances monocyte activation and reprogrammes immune cell metabolism
Source: Front Immunol. 2026 Aug 14;17:1910812. doi: 10.3389/fimmu.2026.1910812 (PMC13521880; doi:10.3389/fimmu.2026.1910812)
Supplement: Supplementary file 1 [file DataSheet1.pdf]

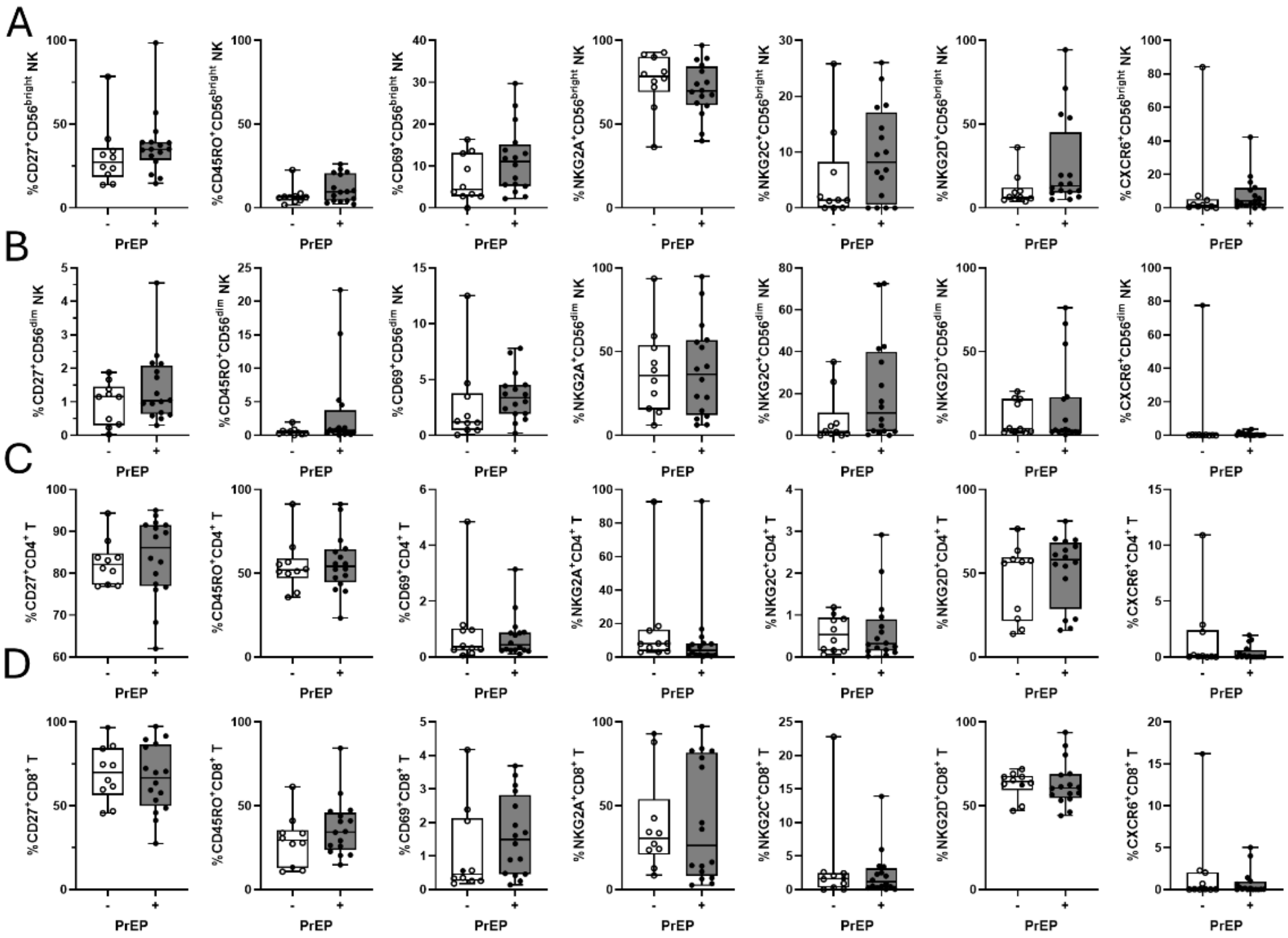

Supplemental Figure 1. Daily PrEP does not alter lymphocyte phenotypic profiles. PBMC were isolated from peripheral blood of PrEP naive individuals (white; n=11) or individuals established on PrEP (grey; n=15) and stained with fluorochrome conjugated antibodies for CD56, CD3, CD4, CD8, CD27, CD45RO, CD69, NKG2C, NKG2D, CXCR6, for flow cytometric analysis. The frequency of **(A)** $CD56^{bright}$ NK cells, **(B)** $CD56^{bright}$ NK cells, **(C)** $CD4^{+}$ T cells, or **(D)** $CD8^{+}$ T cells expressing CD27, CD45RO, CD69, NKG2C, NKG2D or CXCR6. Each dot represents an individual donor, and data were analysed using an unpaired t test; however, results were not statistically significant.

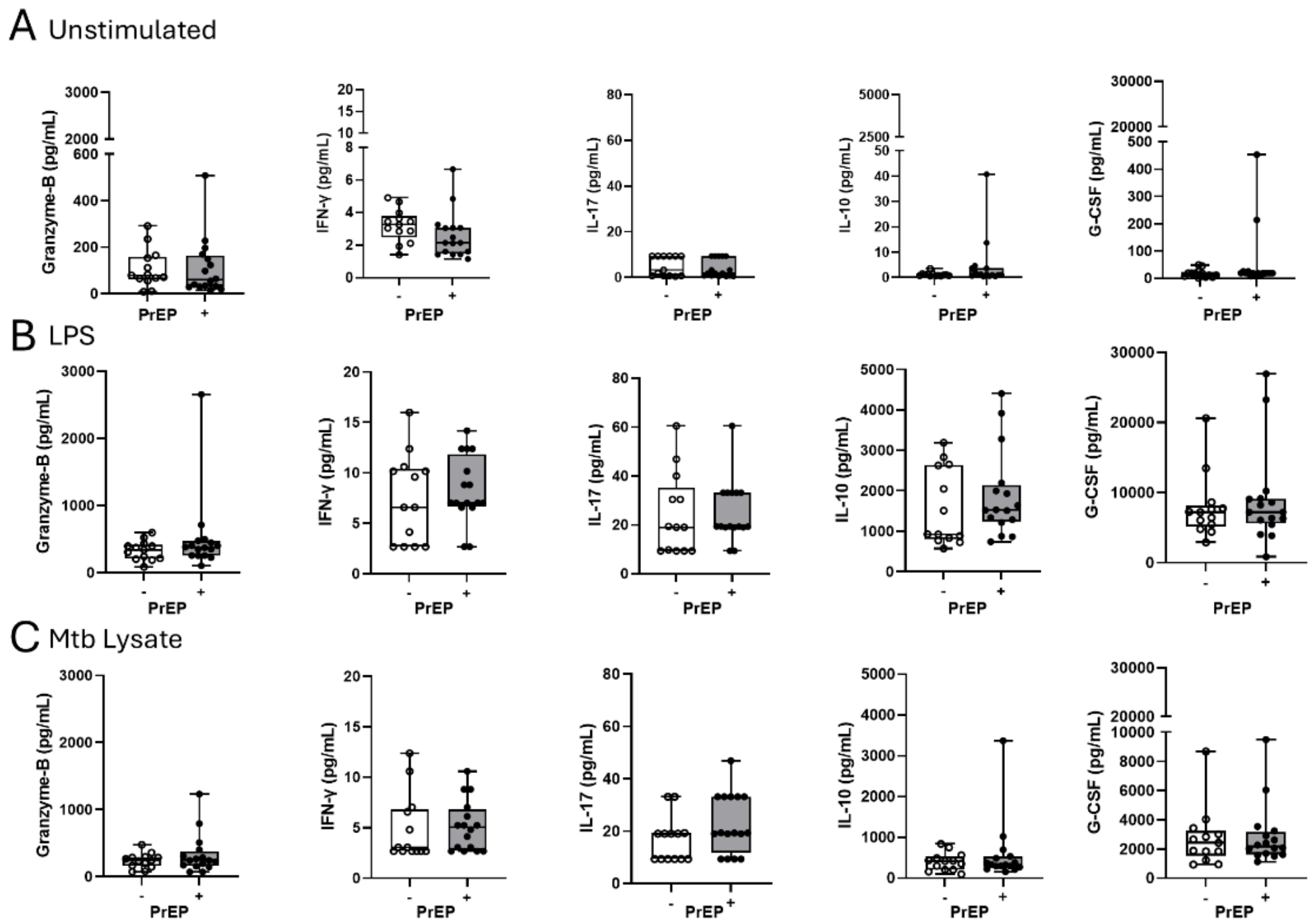

Supplemental Figure 2. Monocyte functional outputs that remain unchanged in individuals established on PrEP. Monocytes were magnetically sorted from the PBMC of PrEP naive individuals (white; n=11) or individuals established on PrEP (grey; n=15) and **(A)** left unstimulated, **(B)** stimulated with LPS (10 ng/ml) or **(C)** stimulated with Mtb lysate (10 µg/ml) for 18 hours. The concentrations (pg/ml) of Granzyme-B, IFN-γ, IL-17, IL-10 or G-CSF in the supernatant were measured using a Luminex Discovery assay. Each dot represents an individual donor, and data were analysed using an unpaired t test; however, results were not statistically significant.

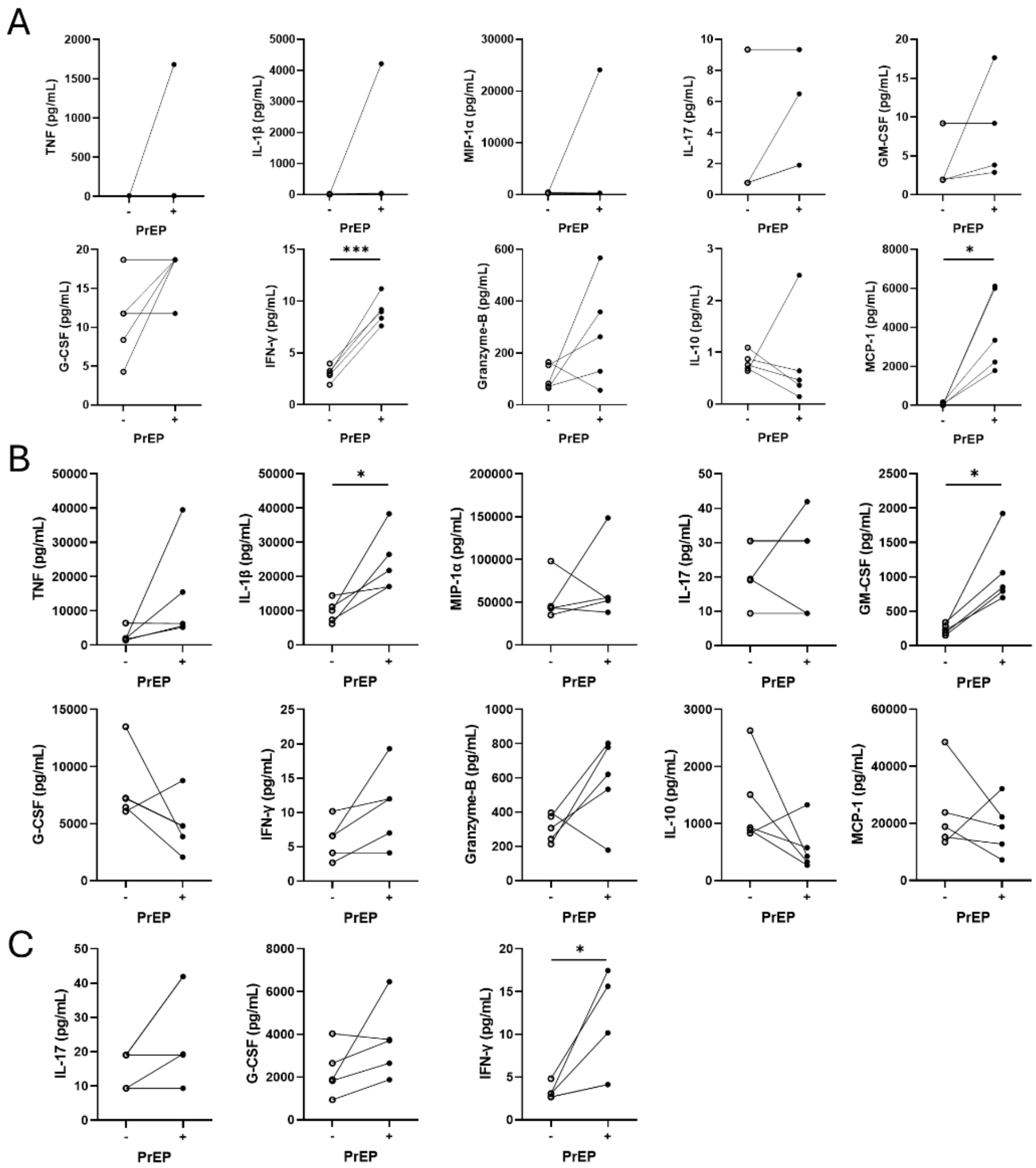

Supplemental Figure 3. The effect of PrEP on cytokine and chemokine production in resting and stimulated monocytes. PBMC were isolated from the matched blood (n=5) of PrEP naïve individuals at T1 (-; open circle) who were established on PrEP at T2 (+; closed circle). Magnetically sorted monocytes were **(A)** left unstimulated, **(B)** stimulated with LPS (10 ng/ml) or **(C)** stimulated with Mtb lysate (10 µg/ml) for 18 hours. The concentrations (pg/ml) of TNF, IL-1β, MIP-1α, IL-17, GM-CSF, G-CSF, IFN-γ, Granzyme-B, IL-10, and MCP-1 in the supernatant were measured using a Luminex Discovery assay. Each dot represents matched data at each timepoint joined by a line. Data were analysed using a two-tailed paired t test where *P<0.05 or ***P<0.001; (A) IFN-γ P= 0.0004, MCP-1 P= 0.0146, (B) IL-1β P= 0.0336, GM-CSF P= 0.0161, (C) IFN-γ P= 0.0256.
